# Heterogeneity in maternal perceptions of psychological birth trauma: a latent profile analysis and predictor study

**DOI:** 10.3389/fpubh.2026.1972281

**Published:** 2026-10-12

**Authors:** Longqiong Wang, Weiwei Wei

**Affiliations:** 1 Department of Obstetrics, The First Affiliated Hospital of Chongqing Medical University, Chongqing, China; 2 Nursing Department, The First Affiliated Hospital of Chongqing Medical University, Chongqing, China

**Keywords:** influencing factors, latent profile analysis, Post-Traumatic Stress Disorder, psychological childbirth trauma perception, puerperium

## Abstract

**Importance:**

The heterogeneity in maternal childbirth trauma perception remains poorly understood, hindering the development of targeted prevention strategies. Identifying distinct risk subgroups and their associated modifiable factors is crucial for informing early interventions aimed at reducing the risk of Postpartum Post-Traumatic Stress Disorder (PP-PTSD).

**Objective:**

To identify heterogeneous classes of maternal perceived childbirth trauma and to examine their cross-sectional correlates and short-term longitudinal associations with the development of PP-PTSD symptoms from 3 to 42 days postpartum.

**Design, setting, and participants:**

This prospective longitudinal study enrolled 300 women following vaginal delivery at a tertiary hospital in Chongqing between February and April 2024. Participants were assessed at two time points: 3 days and 42 days postpartum.

**Main outcomes and measures:**

The primary outcome was childbirth trauma perception, measured using the Birth Trauma Perception Scale for Women During Vaginal Delivery (BTPS-WVD). PP-PTSD symptoms were assessed with the Post-Traumatic Stress Disorder (PTSD) Checklist—Civilian Version (PCL-C). Associated factors examined included perineal pain, breast engorgement, mother-infant separation, and partner support, collected via structured questionnaires.

**Results:**

Latent profile analysis identified four trauma perception classes based on changes in perception scores between the two time points: severe (C1, 13.67%), high (C2, 20.67%), moderate (C3, 48.33%), and low (C4, 17.33%). Factors associated with a lower likelihood of belonging to the high-risk classes (C1/C2) included laboring in shared rooms (OR = 0.362), abnormal fetal heart rate during labor (OR = 0.314), and having the husband as the primary caregiver (OR = 0.410). Factors associated with a higher likelihood included severe breast engorgement affecting mood (OR = 7.084), perineal pain lasting >2 days (OR = 4.019), pain interfering with daily life (OR = 4.899), and mother-infant separation (OR = 4.600). Notably, PTSD symptom scores showed a significant increase from 3 to 42 days postpartum in the severe and high trauma perception classes (C1 and C2) compared to the moderate and low classes (C3 and C4; *P* < 0.05), suggesting that early trauma perception class membership is associated with subsequent symptom worsening.

**Conclusions and relevance:**

Maternal childbirth trauma perception exhibits four distinct classes, characterized by different patterns of early postpartum change. Postpartum physical discomfort and mother-infant separation emerged as key modifiable associated factors, while a supportive intrapartum environment and partner involvement appeared protective. The observed association between high-risk trauma perception classes and subsequent worsening of PTSD symptoms highlights the potential value of early identification. Future targeted interventions focusing on pain management, lactation support, and enhanced partner engagement may be beneficial, though prospective validation of these factors as predictors is warranted.

## Introduction

Childbirth is a significant event in a woman's life, involving not only substantial physiological changes but also complex psychological experiences. Negative childbirth experiences can result in severe psychological trauma for parturients (1). Studies indicate that globally, 20 to 68.6% of parturients experience intense fear, helplessness, or a sense of loss of control during delivery, leading to traumatic birth (2). Some research even suggests that up to 30.5% of obstetricians and gynecologists have personally experienced childbirth trauma (3). Psychological birth trauma (PBT) specifically refers to the subjective distressing feelings triggered by childbirth-related events, manifesting as intertwined emotional experiences that begin during labor and persist into the postpartum period (4).

Traumatic birth not only affects the immediate experience of delivery but also has profound and lasting impacts on postpartum physical and mental health. This trauma exhibits a “ripple effect”, being significantly associated with risks of postpartum post-traumatic stress disorder (PP-PTSD), postpartum depression, and more. It may also interfere with the establishment of the mother-infant relationship by affecting maternal mood and parenting behaviors (such as breastfeeding), posing potential risks to the long-term development of the infant, and even influencing marital relationships and future reproductive decisions (5–8). Notably, childbirth trauma is closely linked to the development of Post-Traumatic Stress Disorder (PTSD). The prevalence of childbirth-related PTSD among all postpartum women is approximately 5%−6%; in the United States alone, about 240,000 women are affected annually, with 18.5 to 41.2% of women reporting PTSD symptoms in cases of complex childbirth (9). A meta-analysis revealed that 4% of postpartum women develop PTSD after a traumatic birth, a proportion that rises to 18.5% in high-risk samples (10). Psychological birth trauma and related PTSD represent a significant disease burden, affecting approximately 6.6 million mothers and 1.7 million fathers or co-parents globally each year (11). Therefore, a deeper understanding of the nature and composition of childbirth trauma perception is crucial for safeguarding maternal and infant health.

PBT and PP-PTSD are conceptually related but distinct phenomena. PBT refers to subjective distressing feelings triggered by childbirth (4), whereas PTSD is a clinical syndrome defined by persistent re-experiencing, avoidance, and hyperarousal. Unlike PTSD, PBT is inherently subjective and context-dependent, shaped by the woman's individual appraisal of threat, control, fear, interpersonal treatment, and unmet expectations. PBT also differs from complex PTSD (CPTSD), which involves broader self-organization disturbances following prolonged trauma. Not all women who experience PBT develop PTSD. Thus, we treat childbirth trauma perception and PTSD symptoms as related but distinct constructs, without implying a necessary progression from one to the other.

However, traditional research often treats the maternal population as a homogeneous whole or focuses solely on single dimensions or total scores of trauma perception. This “variable-centered” paradigm may obscure important heterogeneity within the group. Studies indicate that there are essential differences among parturients in their cognitive, emotional responses, and stress patterns regarding childbirth events, meaning there are potential categories characterized by different symptom profiles (12). For example, individuals with anxiety traits, a lack of positive coping strategies, or a history of domestic violence are more likely to develop severe childbirth trauma (13). Some parturients may primarily exhibit distrust in medical procedures, while others are more troubled by physical pain or lack of social support. Therefore, employing latent profile analysis to identify subgroups with distinct characteristics based on individuals' response patterns across multiple indicators can help provide a basis for the clinical precision identification of high-risk individuals and the implementation of targeted prevention and intervention. Candidate factors were selected based on prior evidence linking childbirth trauma to intrapartum clinical events and postpartum recovery experiences. Intrapartum factors included delivery environment, fetal monitoring events, and companion support, which may influence maternal control and perceived safety. Postpartum factors included perineal pain, breast engorgement, mother-infant separation, and partner support, given their established roles in early maternal adaptation. These factors are further discussed in relation to our findings in the Discussion section.

Person-centered studies have identified heterogeneous PP-PTSD symptom profiles. However, these studies focused on PTSD symptom outcomes rather than on subjective childbirth trauma perception. The present study extends this line of research by using LPA to identify heterogeneous classes of trauma perception—a subjective appraisal of the birth experience—and by examining their longitudinal associations with subsequent PTSD symptoms.

This study aims to use latent profile analysis to explore the potential heterogeneous classes of childbirth trauma perception among parturients at different time points during the puerperium. Secondly, it seeks to identify and compare the differences in demographic characteristics, obstetric clinical features, and postpartum recovery status among parturients in different classes, analyzing their key predictive factors. Finally, it aims to longitudinally investigate the developmental trajectories of early PP-PTSD symptoms among parturients in different trauma perception classes. The innovation of this study lies in being the first, within the Chinese cultural context, to systematically reveal the diverse patterns of childbirth trauma perception among parturients from an individual-centered perspective, with the goal of providing empirical evidence for early clinical risk stratification and precise psychological intervention.

## Methods

### Study design and participants

This prospective longitudinal study enrolled women following vaginal delivery at a tertiary hospital in Chongqing, China, between February and April 2024. Participants were assessed at two time points: 3 days (T1) and 42 days (T2) postpartum.

Inclusion criteria were: gestational age 37–42 weeks, singleton live birth, normal vaginal delivery; no severe obstetric complications (e.g., postpartum hemorrhage); no underlying physical diseases or psychiatric history; and provision of signed informed consent. Exclusion criteria were: severe perineal lacerations (degree III or above); instrumental delivery (forceps or vacuum extraction); neonatal Apgar score at 1 min ≤ 7; communication barriers; failure to complete both questionnaire assessments; questionnaires with missing items or completion time < 120 s. All participants provided written informed consent prior to enrollment. The study was approved by the Ethics Committee of the First Affiliated Hospital of Chongqing Medical University (Ethics No: K2024–082–01). These exclusions reduced confounding and ensured the BTPS-WVD's validity, as the scale was specifically validated for vaginal deliveries. However, this may limit generalizability to women with pre-existing conditions or cesarean/instrumental deliveries.

The sample size was calculated using the principle of 10 events per variable (10 EPV). Based on previous systematic reviews and empirical studies on childbirth trauma, the number of candidate associated factors for childbirth trauma in women with vaginal delivery was estimated to be approximately 15–20, encompassing demographic characteristics, obstetric clinical events, and postpartum recovery variables. Accordingly, the estimated sample size ranged from 150 to 200.

### Study instruments

#### General information questionnaire

A self-designed questionnaire was developed based on the study objectives. All non-scale variables were assessed using self-designed single-item questions. Response categories for sociodemographic variables were: age (< 25, 25–34, ≥35 years), education (junior high or below, high school, college or above), occupation (employed, unemployed), and monthly household income (< 5,000, 5,000–10,000, >10,000 CNY). Clinical variables were assessed as yes/no or categorical responses as presented in Table 1. No response categories were recoded for analysis.

**Table 1:** Coding scheme for independent variables (binary categorical variables).

Independent variable	Coding scheme
Sharing delivery room with other parturients	No = 0; Yes = 1
Decision-maker for abnormalities during labor	Family = 0; Self = 1
Abnormal fetal heart rate during labor	No = 0; Yes = 1
Location during puerperium	Maternity center = 0; Home = 1
Separation from newborn during puerperium	No = 0; Yes = 1
Husband as primary caregiver during puerperium	No = 0; Yes = 1
Occurrence of breast engorgement during puerperium	Yes = 0; No = 1
Breast engorgement severely affecting mood	No = 0; Yes = 1
Perineal wound pain lasting >2 days	No = 0; Yes = 1
Wound pain affecting daily life	No = 0; Yes = 1

All variables were coded as binary indicators based on original questionnaire responses. No response categories were collapsed or recoded.

#### Birth trauma perception scale for women during vaginal delivery (BTPS-WVD)

This scale was developed by Lian Zerong in 2023 (14). It consists of 31 items across 4 dimensions: medical support, labor pain, family support, and childbirth outcome trauma perception. The scale uses a 5-point Likert scale: 1 = “Strongly Disagree', 2 = “Disagree”, 3 = “Neutral”, 4 = “Agree”, 5 = “Strongly Agree”. The total score is the sum of all 31 items, with a higher score indicating a higher perceived level of childbirth trauma. The scale's Cronbach's α coefficient is 0.969.

#### Postpartum Post-Traumatic Stress Disorder (PP-PTSD) scale

The assessment for PP-PTSD utilized the civilian version of the PTSD Checklist (PCL-C), developed by Ruggiero et al. (15). based on the Diagnostic and Statistical Manual of Mental Disorders, Fourth Edition (DSM-IV). The scale comprises 17 items across 3 dimensions: re-experiencing symptoms, avoidance/numbing symptoms, and hyperarousal symptoms. It is widely used in clinical research on maternal PTSD. Items are rated on a 5-point Likert scale (1–5 points) based on symptom severity, yielding a total score ranging from 17 to 85. A higher score indicates a greater likelihood of PTSD, with a total score ≥ 38 indicating a positive screen for PTSD, but this does not constitute a clinical diagnosis. The PCL-C was used as a measure of PTSD symptom severity rather than as a diagnostic tool. The scale's Cronbach's α coefficient is 0.931. Yang Xiaoyun translated and adapted the scale into Chinese in 2007 (16). Research by Wang Meifang supports the use of the PCL-C in Chinese populations (17). Wang Xinling has previously employed this scale to measure the incidence of postpartum PTSD (18). The PCL-C was selected because this study examined associations with PTSD symptom severity rather than clinical diagnoses, and it has been widely used with adequate psychometric properties in Chinese perinatal populations. The City BiTS, a DSM-5-based childbirth-specific measure, was not yet widely available in a validated Chinese version when this study was initiated. We acknowledge this limitation and have noted it in the Discussion.

### Survey procedure

The questionnaire items were converted into an electronic form using the “Wenjuanxing” platform. Participants accessed and completed the questionnaires at two time points—3 days (T1) and 42 days (T2) postpartum—by scanning a QR code with WeChat, and submissions were sent to the platform's backend. All questionnaires were self-administered, and no compensation was provided. The researcher personally distributed the questionnaires, using standardized instructions and adhering to the principle of informed consent. At T2, up to two reminders were sent via WeChat at 3-day intervals to non-responders. A total of 310 questionnaires were collected at T1. After excluding four questionnaires with completion times of less than 120 s, 300 valid questionnaires remained, resulting in an effective response rate of 98%. All 300 participants who completed the T1 assessment were followed up and reassessed at T2.

### Statistical analysis

#### Latent profile analysis (LPA)

To identify heterogeneous classes of childbirth trauma perception, we first calculated change scores for each of the four BTPS-WVD dimensions (medical support, labor pain, family support, childbirth outcome) and the total score by subtracting the T1 (3 days postpartum) score from the T2 (42 days postpartum) score. These change scores were then standardized and entered as indicators into the LPA. The model parameters were estimated using the maximum likelihood method, and each participant was assigned to the class with the maximum posterior probability.

Model fit was evaluated using the Bayesian Information Criterion (BIC), Akaike Information Criterion (AIC), and the Sample-Size Adjusted BIC (aBIC). The number of latent classes was considered appropriate when the average posterior probability for each class was >0.7 and the Entropy index was >0.8. A K-class model was deemed to have a better fit than a K-1 class model if the *p*-value for the Bootstrapped Likelihood Ratio Test (BLRT) or the Vuong-Lo-Mendell-Rubin Likelihood Ratio Test (LMRT) was < 0.05. Model selection was guided by a combination of statistical criteria and practical considerations. In addition to fit indices (AIC, BIC, aBIC) and likelihood-ratio tests (BLRT, LMRT), we evaluated each candidate model based on class size (requiring each class to contain >5% of the sample), entropy (>0.80), interpretability of the profiles, and theoretical meaningfulness, rather than relying on any single statistical test alone. This analysis was performed using Mplus version 8.3.

#### General statistical analysis and association analyses

Normally distributed continuous data are presented as mean ± standard deviation. Group comparisons were conducted using analysis of variance (ANOVA), with *post-hoc* pairwise comparisons performed using the SNK-q test. Comparisons between the two time points were made using the paired *t*-test. Non-normally distributed continuous data are presented as median and interquartile range. Group comparisons were conducted using the Kruskal–Wallis test, with *post-hoc* pairwise comparisons adjusted using the Dunn-Bonferroni method. Comparisons between the two time points were made using the Wilcoxon signed-rank test. Categorical data are presented as frequency and percentage, and group comparisons were conducted using the Chi-square test or Fisher's exact test, as appropriate.

To examine factors associated with latent class membership, variables with a *p*-value < 0.05 in univariate analyses were entered as candidate associated factors into an unordered multinomial multivariate logistic regression model. Given the data-driven nature of this variable selection procedure, we acknowledge that it may produce unstable estimates and increase the risk of Type I error. Thus, the results of this exploratory analysis should be interpreted with caution. In addition to *p*-values and odds ratios, 95% confidence intervals were reported for all estimates, and greater emphasis was placed on the precision and magnitude of the associations rather than on significance thresholds alone. A total of 10 candidate variables were entered into the multivariate model based on their significance in univariate analyses (*p* < 0.05). Of note, the categorical variables entered into the multivariate regression model (Table 1) were coded as binary indicators based on the original questionnaire response options. No response categories were collapsed or recoded for analysis.

To explore the short-term longitudinal association between latent class membership and changes in PP-PTSD symptoms, we compared PCL-C scores across classes at both T1 and T2, and tested the class-by-time interaction using repeated-measures ANOVA. This part of the analysis was performed using SAS version 9.4 (Copyright © 2016 SAS Institute Inc., Cary, NC, USA). A two-tailed *p*-value < 0.05 was considered statistically significant.

It should be noted that the change scores (T2–T1) entered into the LPA contain information about both the baseline level at T1 and the direction of change over time. Thus, the resulting classes reflect a combination of the initial severity of trauma perception and its subsequent evolution. Accordingly, class labels such as “severe,” “high,” “moderate,” and “low” are used to describe the overall level of trauma perception characterized by each class, while the temporal change patterns are also presented and discussed. Importantly, because the latent classes were derived from change scores between T1 and T2, the subsequent analysis of PTSD changes across these same classes over the same interval should be interpreted as an exploratory association, not as prospective prediction. The non-independence between class derivation and outcome assessment precludes causal or predictive claims.

## Results

This study included 300 postpartum women, with a mean age of 30.08 ± 3.57 years. 83.0% had a bachelor's degree or above, 92.3% were employees or managers, and 94.7% had medical insurance. During pregnancy, 71.7% continued working and 60.0% exercised regularly; 92.0% had limited knowledge of delivery modes. During labor, 62.7% received analgesia, 64.3% received doula support, and 49.3% experienced episiotomy or second-degree perineal laceration; abnormal fetal heart rate occurred in 52.7% and labor intervention in 22.7%. Neonatal gestational age was 39.26 ± 0.90 weeks, birth weight was 3,192.67 ± 334.10 g, and Apgar score was 10 (9, 10). During the postpartum period, 58.7% practiced breastfeeding, 84.3% stayed at home, and 73.0% identified their husband as the primary caregiver; perineal wound pain lasting >2 days was reported by 74.0%, and 40.3% reported that the wound affected daily life (see Table 2).

**Table 2:** Association between maternal characteristics and latent classes of trauma perception.

Variables	Total	Class 1 (*n* = 41)	Class 2 (*n* = 62)	Class 3 (*n* = 145)	Class 4 (*n* = 52)	*t*/*Z*/χ^2^	*P*
Sociodemographic information							
Age, years (mean ± SD)	30.08 ± 3.57	30.24 ±3.55	30.02 ± 3.99	30.15 ± 3.49	29.83 ± 3.36	0.141	0.935
Education level							
College diploma or below	51 (17.00)	4 (9.76)	16 (25.81)	20 (13.79)	11 (21.15)	6.625	0.085
Bachelor's degree or above	249 (83.00)	37 (90.24)	46 (74.19)	125 (86.21)	41 (78.85)		
Able to control own income							
Yes	290 (96.67)	39 (95.12)	61 (98.39)	139 (95.86)	51 (98.08)	/	0.768
No	10 (3.33)	2 (4.88)	1 (1.61)	6 (4.14)	1 (1.92)		
Medical insurance							
No	16 (5.33)	1 (2.44)	5 (8.06)	6 (4.14)	4 (7.69)	/	0.473
Yes	284 (94.67)	40 (97.56)	57 (91.94)	139 (95.86)	48 (92.31)		
Occupation							
Unemployed	23 (7.67)	1 (2.44)	6 (9.68)	14 (9.66)	2 (3.85)	/	0.425
Employee	159 (53.00)	19 (46.34)	31 (50.00)	80 (55.17)	29 (55.77)		
Managerial/professional	118 (39.33)	21 (51.22)	25 (40.32)	51 (35.17)	21 (40.38)		
Continued working during pregnancy							
Yes	215 (71.67)	29 (70.73)	38 (61.29)	107 (73.79)	41 (78.85)	4.948	0.176
No	85 (28.33)	12 (29.27)	24 (38.71)	38 (26.21)	11 (21.15)		
Exercised regularly during pregnancy							
Yes	180 (60.00)	25 (60.98)	35 (56.45)	91 (62.76)	29 (55.77)	1.189	0.756
No	120 (40.00)	16 (39.02)	27 (43.55)	54 (37.24)	23 (44.23)		
Knowledge level of delivery modes							
Completely unaware	24 (8.00)	4 (9.76)	3 (4.84)	9 (6.21)	8 (15.38)	/	0.061
Very little	190 (63.33)	19 (46.34)	45 (72.58)	96 (66.21)	30 (57.69)
Some understanding	86 (28.67)	18 (43.90)	14 (22.58)	40 (27.59)	14 (26.92)
Gravidity/Parity (GP)							
1	219 (73.00)	33 (80.49)	45 (72.58)	102 (70.34)	39 (75.00)	1.796	0.616
≥2	81 (27.00)	8 (19.51)	17 (27.42)	43 (29.66)	13 (25.00)		
Labor process							
Total duration of labor, min [M (p25, p75)]	502.50 (315.50, 823)	495 (409, 868)	676 (325, 845)	472 (287, 735)	583 (294, 906.50)	5.300	0.151
Labor analgesia							
No	112 (37.33)	11 (26.83)	18 (29.03)	63 (43.45)	20 (38.46)	6.105	0.107
Yes	188 (62.67)	30 (73.17)	44 (70.97)	82 (56.55)	32 (61.54)		
Doula during labor							
No	107 (35.67)	8 (19.51)	25 (40.32)	55 (37.93)	19 (36.54)	5.590	0.133
Yes	193 (64.33)	33 (80.49)	37 (59.68)	90 (62.07)	33 (63.46)		
Perineal condition							
Intact/Idegree	128 (42.67)	21 (51.22)	26 (41.94)	60 (41.38)	21 (40.38)	/	0.645
Median episiotomy	24 (8.00)	3 (7.32)	2 (3.23)	14 (9.66)	5 (9.62)
Lateral episiotomy/II degree	148 (49.33)	17 (41.46)	34 (54.84)	71 (48.97)	26 (50.00)
Sharing delivery room with other parturients							
Yes	221 (73.67)	19 (46.34)	49 (79.03)	114 (78.62)	39 (75.00)	18.583	< 0.001
No	79 (26.33)	22 (53.66)	13 (20.97)	31 (21.38)	13 (25.00)		
Psychological discomfort from sharing room							
Yes	28 (9.33)	3 (7.32)	11 (17.74)	11 (7.59)	3 (5.77)	/	0.119
No	272 (90.67)	38 (92.68)	51 (82.26)	134 (92.41)	49 (94.23)		
Feeling disrespected/offended during delivery							
Yes	70 (23.33)	8 (19.51)	18 (29.03)	37 (25.52)	7 (13.46)	4.680	0.197
No	230 (76.67)	33 (80.49)	44 (70.97)	108 (74.48)	45 (86.54)		
Decision-maker for abnormalities during labor							
Self	269 (89.67)	39 (95.12)	48 (77.42)	132 (91.03)	50 (96.15)	14.008	< 0.001
Family member	31 (10.33)	2 (4.88)	14 (22.58)	13 (8.97)	2 (3.85)		
Labor intervention							
Yes	68 (22.67)	7 (17.07)	17 (27.42)	27 (18.62)	17 (32.69)	5.867	0.118
No	232 (77.33)	34 (82.93)	45 (72.58)	118 (81.38)	35 (67.31)		
Abnormal fetal heart rate during labor							
Yes	158 (52.67)	13 (31.71)	38 (61.29)	79 (54.48)	28 (53.85)	9.295	0.026
No	142 (47.33)	28 (68.29)	24 (38.71)	66 (45.52)	24 (46.15)		
Number of vaginal examinations							
1~3	158 (52.67)	21 (51.22)	28 (45.16)	88 (60.69)	21 (40.38)	10.651	0.100
4~7	115 (38.33)	17 (41.46)	30 (48.39)	44 (30.34)	24 (46.15)		
>7	27 (9.00)	3 (7.32)	4 (6.45)	13 (8.97)	7 (13.46)		
Duration in delivery room before birth							
1–5 h	161 (53.67)	21 (51.22)	26 (41.94)	88 (60.69)	26 (50.00)	9.654	0.140
6–10 h	89 (29.67)	16 (39.02)	23 (37.10)	35 (24.14)	15 (28.85)		
>10 h	50 (16.67)	4 (9.76)	13 (20.97)	22 (15.17)	11 (21.15)
Delivery outcome							
Gestational weeks (mean ± SD)	39.26 ± 0.90	39.47 ± 0.60	39.29 ± 1.02	39.20 ± 0.91	39.25 ± 0.93	0.949	0.417
Apgar score [M (p25, p75)]	10 (9, 10)	10 (9, 10)	10 (9, 10)	10 (9, 10)	10 (9, 10)	1.654	0.647
Neonatal weight, g (mean ± SD)	3192.67 ± 334.10	3208.54 ± 328.27	3174.03 ± 354.74	3195.14 ± 334.09	3195.48 ± 231.85	0.098	0.961
Postpartum blood loss, ml [M (p25, p75)]	260 (220, 300)	260 (220, 330)	260 (230, 320)	250 (210, 290)	260 (230, 350)	3.536	0.316
Neonatal sex							
Male	156 (52.00)	19 (46.34)	32 (51.61)	81 (55.86)	24 (46.15)	2.108	0.550
Female	144 (48.00)	22 (53.66)	30 (48.39)	64 (44.14)	28 (53.85)
Puerperium information							
Infant feeding method							
Breastfeeding	176 (58.67)	24 (58.54)	34 (54.84)	81 (55.86)	37 (71.15)	/	0.374
Mixed feeding	113 (37.67)	17 (41.46)	25 (40.32)	58 (40.00)	13 (25.00)		
Formula feeding	11 (3.67)	0 (0.00)	3 (4.84)	6 (4.14)	2 (3.85)		
Location during puerperium							
Maternity center	47 (15.67)	2 (4.88)	16 (25.81)	21 (14.48)	8 (15.38)	8.594	0.035
Home	253 (84.33)	39 (95.12)	46 (74.19)	124 (85.52)	44 (84.62)		
Separation from newborn during puerperium							
Yes	55 (18.33)	17 (41.46)	8 (12.90)	20 (13.79)	10 (19.23)	17.896	< 0.001
No	245 (81.67)	24 (58.54)	54 (87.10)	125 (86.21)	42 (80.77)		
Husband as primary caregiver during puerperium							
Yes	219 (73.00)	39 (95.12)	36 (58.06)	108 (74.48)	36 (69.23)	17.733	< 0.001
No	81 (27.00)	2 (4.88)	26 (41.94)	37 (25.52)	16 (30.77)		
Occurrence of breast engorgement during puerperium							
No	226 (75.33)	24 (58.54)	53 (85.48)	110 (75.86)	39 (75.00)	9.688	0.021
Yes	74 (24.67)	17 (41.46)	9 (14.52)	35 (24.14)	13 (25.00)		
Breast engorgement severely affecting mood							
Yes	45 (15.00)	6 (14.63)	21 (33.87)	14 (9.66)	4 (7.69)	22.748	< 0.001
No	255 (85.00)	35 (85.37)	41 (66.13)	131 (90.34)	48 (92.31)		
Perineal wound pain lasting >2 days							
Yes	222 (74.00)	37 (90.24)	54 (87.10)	100 (68.97)	31 (59.62)	18.653	< 0.001
No	78 (26.00)	4 (9.76)	8 (12.90)	45 (31.03)	21 (40.38)		
Wound pain affecting daily life							
Yes	121 (40.33)	30 (73.17)	26 (41.94)	51 (35.17)	14 (26.92)	23.927	< 0.001
No	179 (59.67)	11 (26.83)	36 (58.06)	94 (64.83)	38 (73.08)		

**I**ndicates that Fisher's exact test was performed due to small expected cell counts, with no statistical significance.

To address the study objectives, we first examined overall changes in childbirth trauma perception from T1 to T2. We then applied latent profile analysis (LPA) to identify heterogeneous classes of trauma perception based on individual change patterns. Finally, we compared class membership with maternal characteristics and subsequent PTSD symptom changes to explore associated factors and potential links to postpartum psychological outcomes.

### Changes in childbirth trauma perception

Overall, the total scores across all dimensions of childbirth trauma perception at 42 days postpartum showed a significant increase compared to those at 3 days postpartum (*P* < 0.05). However, as shown in Figure 1, the individual change trajectories exhibited heterogeneity. Specifically, for some women, the total trauma perception score at 42 days postpartum showed a decreasing trend compared to the 3 days assessment, while for others it showed an increasing trend. The trends in score changes are illustrated in Figure 1, and the specific scores are detailed in Table 3.

**Figure 1:**
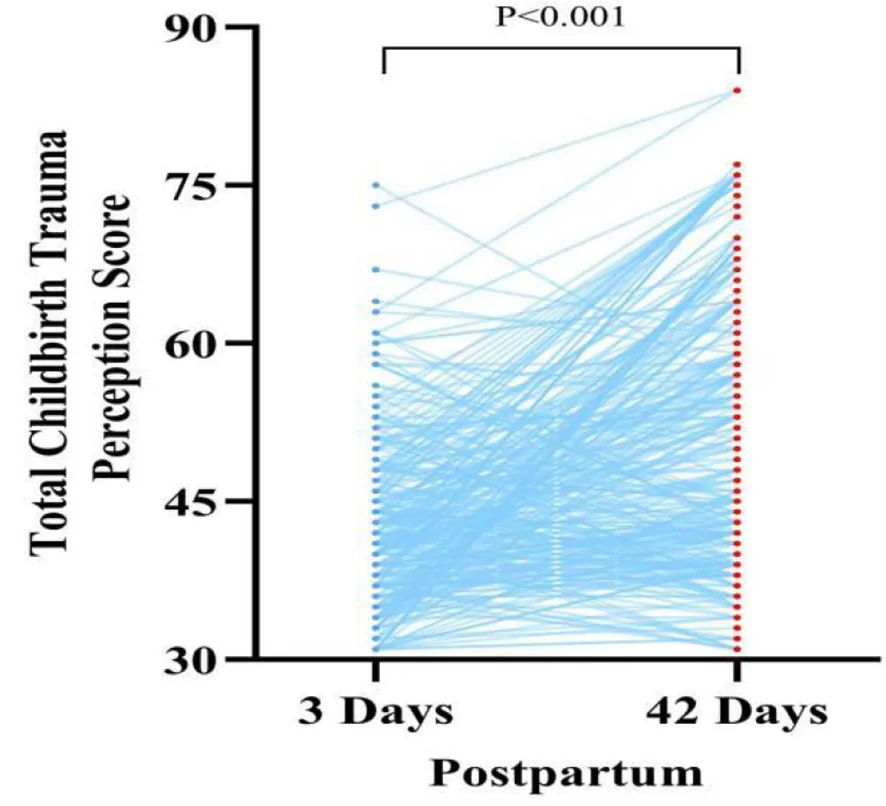
Temporal changes in childbirth trauma perception.

**Table 3:** Comparison of childbirth trauma perception at 3 days and 42 days postpartum.

Childbirth trauma perception	3 days postpartum	42 days postpartum	Difference	*t*	*P*
Medical support trauma perception	15.51 ± 2.78	18.38 ± 5.17	2.87 ± 5.42	9.157	< 0.001
Labor pain trauma perception	12.83 ± 4.37	16.09 ± 5.98	3.26 ± 6.82	8.278	< 0.001
Family support trauma perception	6.36 ± 0.88	7.11 ± 1.75	0.75 ± 1.81	7.160	< 0.001
Childbirth outcome trauma perception	7.79 ± 0.04	9.64 ± 4.09	1.85 ± 4.61	6.956	< 0.001
Total score	42.49 ± 8.00	51.21 ± 13.08	8.72 ± 14.00	10.789	< 0.001

#### Results of latent profile analysis (LPA)

A total of six latent class models with different numbers of profiles were fitted. The model fit indices are presented in Table 4. As the number of latent classes increased, the AIC, BIC, and aBIC values decreased. When considering models beyond four classes, the five-class model showed a non-significant LMR test (*p* > 0.05), and the six-class model contained two very small classes (*n* = 12 and *n* = 10), suggesting over-extraction. Considering the statistical indices together with class size, interpretability, and parsimony, the four-class solution was retained as the most appropriate. The four-class model comprised the following groups: C1 (*n* = 41, 13.67%), C2 (*n* = 62, 20.67%), C3 (*n* = 145, 48.33%), and C4 (*n* = 52, 17.33%). The average posterior probabilities for individuals assigned to their respective latent classes were 0.96, 0.94, 0.94, and 0.92, indicating high reliability in the classification results of the four-class model.

**Table 4:** Fit indices of latent class models for maternal childbirth trauma perception.

Latent classes number	Loglikelihood	BIC	AIC	aBIC	Entropy	BLRT	LMRT	Class Sie *n* (%)	Average posterior probability
1	−2125.904	4271.807	4308.845	4277.131	—	—	—	C1 = 300 (100%)	C11 = 0.00
2	−1837.294	3706.588	3765.849	3715.106	0.923	< 0.001	0.008	C12 = 09 (69.67%)/ C29 = 1 (30.33%)	C10 = 0.98/C20 = 0.97
3	−1770.728	3585.457	3666.940	3597.169	0.837	< 0.001	0.045	C16 = 4 (21.33%)/ C21 = 54 (51.33%)/ C38 = 2 (27.33%)	C10 = 0.90/C20 = 0.92/C30 = 0.96
4	−1707.668	3471.335	3575.041	3486.242	0.888	< 0.001	0.018	C14 = 1 (13.67%)/ C26 = 2 (20.67%)/ C31 = 45 (48.33%)/ C45 = 2 (17.33%)	C10 = 0.96/C20 = 0.94/C30 = 0.94/C40 = 0.92
5	−1659.044	3386.088	3512.017	3404.189	0.906	< 0.001	0.177	C15 = 2 (17.33%)/ C21 = 42 (47.33%)/ C38 = (2.67%)/ C46 = 0 (20.00%)/ C53 = 8 (12.67%)	C10 = 0.92/C20 = 0.94/C31 = 0.00/C40 = 0.93/C50 = 0.97
6	−1627.190	3334.380	3482.531	3355.674	0.911	< 0.001	0.005	C15 = 2 (17.33%)/ C25 = 6 (18.67%)/ C31 = 2 (4.00%)/ C43 = 8 (12.67%)/ C51 = 32 (44.00%)/ C61 = 0 (3.33%)	C10 = 0.92/ C20 = 0.92/ C30 = 0.95/ C40 = 0.97/ C50 = 0.94/ C60 = 0.99

The distribution of estimated values from the LPA is shown in Figure 2, and comparisons of actual scores across latent classes are presented in Table 5. Although the LPA was based on change scores (T2–T1), the resulting classes exhibited distinct absolute score levels at both time points (see Table 5). Group C1 scored significantly higher than other groups on Labor Pain, Childbirth Outcome, and the Total Score, and was therefore labeled the “Severe Childbirth Trauma Group”. Groups C2 and C3 scored around the population average on Medical Support, Labor Pain, Family Support, Childbirth Outcome, and the Total Score. Consequently, C2 was labeled the “High Trauma Perception Group” and C3 the “Moderate Trauma Perception Group”. Group C4 had the lowest scores across all dimensions (Medical Support, Labor Pain, Family Support, Childbirth Outcome) and the Total Score, and was labeled the “Low Childbirth Trauma Group”. Of note, these class labels reflect the overall level of trauma perception, while the underlying change patterns are reflected in the score differences between the two time points (see Table 5).

**Figure 2:**
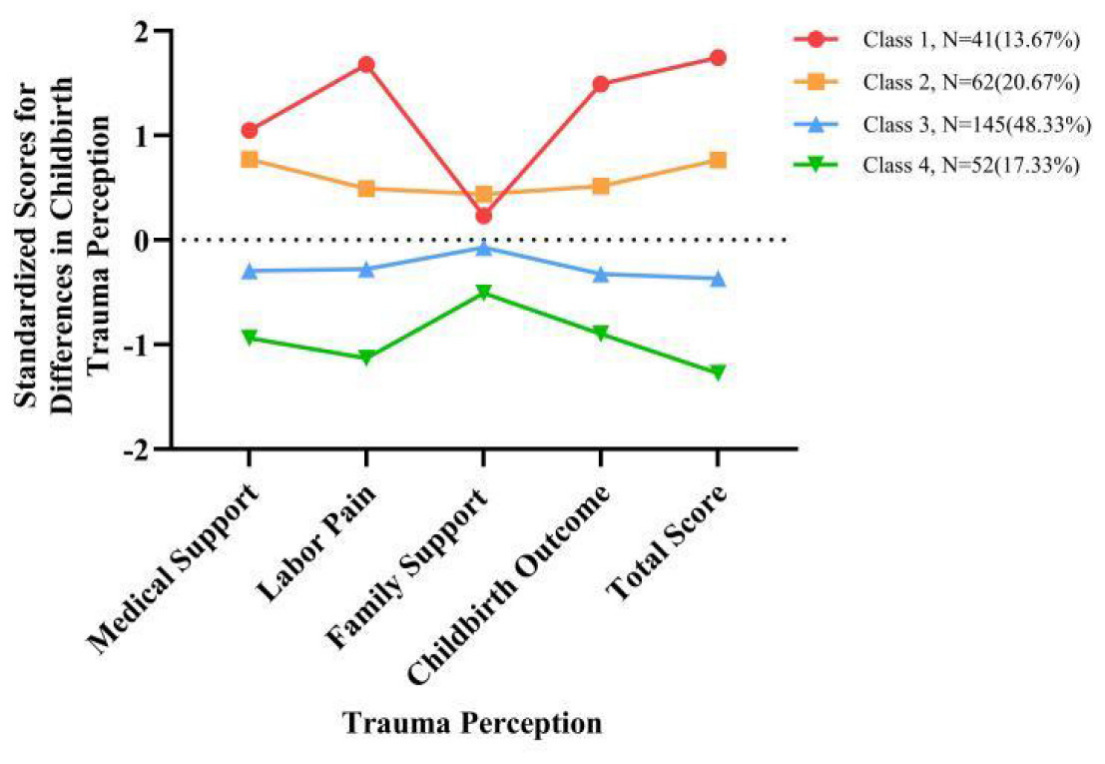
Profile plot of the four latent classes of maternal childbirth trauma perception.

**Table 5:** Association between latent classes of maternal childbirth trauma perception and their dimensions/total scores.

Childbirth trauma perception	Class 1 (*n* = 41)	Class 2 (*n* = 62)	Class 3 (*n* = 145)	Class 4 (*n* = 52)	*F*	*P*
3 days postpartum						
Medical support trauma perception	14.68 ± 1.46	15.34 ± 1.81	15.10 ± 1.94	17.50 ± 4.89^a, b, c^	12.544	< 0.001
Labor pain trauma perception	10.05 ± 3.29	12.74 ± 4.17^a^	12.59 ± 4.19^*a*^	15.81 ± 4.20^a, b, c^	15.805	< 0.001
Family support trauma perception	6.20 ± 0.51	6.40 ± 0.88	6.28 ± 0.81	6.671 ± 1.18^a, c^	3.243	0.022
Childbirth outcome trauma perception	5.90 ± 2.12	7.842 ± 2.69^a^	7.75 ± 3.02^*a*^	9.31 ± 3.33^a, b, c^	10.514	< 0.001
Total score	36.83 ± 5.19	42.32 ± 6.89^a^	41.73 ± 7.39^a^	49.29 ± 8.31^a, b, c^	24.437	< 0.001
42 days postpartum						
Medical support trauma perception	23.00 ± 5.26	22.77 ± 5.63	16.34 ± 3.14^a, b^	15.15 ± 2.46^*a, b*^	66.450	< 0.001
Labor pain trauma perception	24.90 ± 3.43	19.34 ± 4.23^a^	13.94 ± 4.31^a, b^	11.27 ± 3.80^a, b, c^	113.338	< 0.001
Family support trauma perception	7.34 ± 1.67	7.97 ± 2.40^a^	6.94 ± 1.53^b^	6.38 ± 0.80^a, b^	9.425	< 0.001
Childbirth outcome trauma perception	14.73 ± 2.84	11.92 ± 3.89^a^	8.17 ± 3.01^a, b^	6.98 ± 2.72^a, b, c^	69.581	< 0.001
Total score	69.98 ± 5.82	62.00 ± 88.59^a^	45.39 ± 8.11^a, b^	39.79 ± 6.93^a, b, c^	185.129	< 0.001
Difference						
Medical support trauma perception	8.32 ± 4.53	7.44 ± 55.46	1.24 ± 2.72^a, b^	−2.35 ± 3.91^a, b, c^	94.832	< 0.001
Labor pain trauma perception	14.85 ± 3.98	6.60 ± 4.31^a^	1.35 ± 3.35^a, b^	−4.54 ± 3.54^a, b, c^	242.780	< 0.001
Family support trauma perception	1.15 ± 1.61	1.56 ± 2.42	0.66 ± 1.50^b^	−0.29 ± 1.36^a, b, c^	11.754	< 0.001
Childbirth outcome trauma perception	8.83 ± 3.09	4.08 ± 3.49^a^	0.42 ± 2.94^a, b^	−2.33 ± 3.03^a, b, c^	122.119	< 0.001
Total score	33.15 ± 4.67	19.68 ± 4.62^a^	3.66 ± 4.18^a, b^	−9.50 ± 4.49^a, b, c^	913.381	< 0.001

Pairwise comparisons were conducted using the SNK-q test.

^a^*P* < 0.05 compared with Class 1; ^b^*P* < 0.05 compared with Class 2; ^c^*P* < 0.05 compared with Class 3; difference = 42 days postpartum-3 days postpartum. The “difference” row reflects the change scores (T2–T1) that were used as indicators in the LPA.

#### Association between maternal characteristics and latent classes of trauma perception

Significant differences (*P* < 0.05) were observed across the latent classes of trauma perception in the following maternal characteristics: sharing the delivery room with other parturients during labor and delivery, decision-maker in case of abnormalities during delivery, abnormal fetal heart rate during labor, location during the puerperium, separation from the newborn during the puerperium, husband as the primary caregiver during the puerperium, occurrence of breast engorgement during the puerperium, breast engorgement severely affecting mood, perineal wound pain lasting >2 days, and wound pain affecting daily life. The detailed results are presented in Table 2.

#### Results of the multivariate regression model for latent classes of maternal trauma perception

Variables with a significance level of *P* < 0.05 in univariate analysis were included in the multivariate regression model. The coding scheme for independent variables is presented in Table 1. The results of the multinomial multivariate logistic regression model, using C4 as the reference category, showed that sharing the delivery room with other parturients during labor and delivery (OR = 0.362, 95% CI: 0.16–0.81), and abnormal fetal heart rate during labor (OR = 0.314, 95% CI: 0.12–0.72) were negatively associated with C1. Breast engorgement severely affecting mood (OR = 7.084, 95% CI: 2.89–17.35) and perineal wound pain lasting >2 days (OR = 4.019, 95% CI: 1.72–9.39) were identified as factors associated with higher likelihood of belonging to both C1 and C2. When using C3 as the reference category, sharing the delivery room with other parturients during labor and delivery, and abnormal fetal heart rate during labor remained negatively associated with C1. Separation from the newborn during the puerperium and wound pain affecting daily life were identified as factors associated with higher likelihood of belonging to C1. Having the husband as the primary caregiver during the puerperium was negatively associated with C2, while breast engorgement severely affecting mood and perineal wound pain lasting >2 days were factors associated with higher likelihood of belonging to C2. When using C2 as the reference category, abnormal fetal heart rate during labor was negatively associated with C1. Separation from the newborn during the puerperium, having the husband as the primary caregiver during the puerperium, and wound pain affecting daily life were identified as factors associated with higher likelihood of belonging to C1. Details are provided in Table 6.

**Table 6:** Results of the multinomial multivariate logistic regression model for latent classes of maternal trauma perception (OR and 95% CI).

Variable	β	SE	Wald χ2	*P*	OR (95%CI)
C1 vs. C4					
Sharing the delivery room with other parturients during labor and delivery	−1.016	0.510	3.959	0.047	0.362 (0.133, 0.985)
Abnormal fetal heart rate during labor	−1.159	0.490	5.581	0.018	0.314 (0.120, 0.821)
Separation from the newborn during the puerperium	0.881	0.541	2.658	0.103	2.414 (0.837, 6.963)
Husband as the primary caregiver during the puerperium	1.401	0.825	2.887	0.089	4.060 (0.806, 20.442)
Breast engorgement severely affecting mood	1.200	0.760	2.490	0.115	3.319 (0.748, 14.725)
Perineal wound pain lasting >2 days	1.391	0.655	4.510	0.034	4.019 (1.113, 14.512)
Wound pain affecting daily life	1.589	0.510	9.698	0.002	4.899 (1.802, 13.317)
C2 vs. C4					
Sharing the delivery room with other parturients during labor and delivery	−0.162	0.485	0.112	0.738	0.850 (0.329, 2.199)
Abnormal fetal heart rate during labor	0.324	0.410	0.621	0.431	1.382 (0.618, 3.090)
Separation from the newborn during the puerperium	−0.645	0.553	1.362	0.243	0.525 (0.178, 1.550)
Husband as the primary caregiver during the puerperium	−0.696	0.443	2.467	0.116	0.499 (0.209, 1.188)
Breast engorgement severely affecting mood	1.958	0.632	9.605	0.002	7.084 (2.054, 24.437)
Perineal wound pain lasting >2 days	1.767	0.509	12.052	0.001	5.852 (2.158, 15.866)
Wound pain affecting daily life	0.416	0.445	0.872	0.350	1.515 (0.633, 3.626)
C3 vs. C4					
Sharing the delivery room with other parturients during labor and delivery	0.251	0.390	0.415	0.520	1.286 (0.598, 2.763)
Abnormal fetal heart rate during labor	0.022	0.329	0.005	0.946	1.023 (0.537, 1.949)
Separation from the newborn during the puerperium	−0.462	0.432	1.143	0.285	0.630 (0.270, 1.470)
Husband as the primary caregiver during the puerperium	0.195	0.370	0.278	0.598	1.216 (0.589, 2.511)
Breast engorgement severely affecting mood	0.155	0.615	0.064	0.801	1.168 (0.350, 3.898)
Perineal wound pain lasting >2 days	0.389	0.351	1.229	0.268	1.475 (0.742, 2.933)
Wound pain affecting daily life	0.289	0.378	0.586	0.444	1.336 (0.636, 2.803)
C1 vs. C3					
Sharing the delivery room with other parturients during labor and delivery	−1.267	0.435	8.480	0.004	0.282 (0.120, 0.661)
Abnormal fetal heart rate during labor	−1.181	0.432	7.484	0.006	0.307 (0.132, 0.715)
Separation from the newborn during the puerperium	1.343	0.464	8.376	0.004	3.832 (1.543, 9.518)
Husband as the primary caregiver during the puerperium	1.206	0.786	2.356	0.125	3.340 (0.716, 15.583)
Breast engorgement severely affecting mood	1.045	0.603	2.999	0.083	2.842 (0.871, 9.271)
Perineal wound pain lasting >2 days	1.002	0.611	2.688	0.101	2.725 (0.822, 9.031)
Wound pain affecting daily life	1.299	0.435	8.939	0.003	3.667 (1.565, 8.596)
C2 vs. C3					
Sharing the delivery room with other parturients during labor and delivery	−0.413	0.406	1.035	0.309	0.661 (0.298, 1.467)
Abnormal fetal heart rate during labor	0.301	0.336	0.803	0.370	1.351 (0.699, 2.612)
Separation from the newborn during the puerperium	−0.183	0.482	0.144	0.705	0.833 (0.324, 2.143)
Husband as the primary caregiver during the puerperium	−0.891	0.361	6.085	0.014	0.410 (0.202, 0.833)
Breast engorgement severely affecting mood	1.803	0.435	17.192	< 0.001	6.067 (2.587, 14.227)
Perineal wound pain lasting >2 days	1.378	0.451	9.353	0.002	3.967 (1.640, 9.594)
Wound pain affecting daily life	0.126	0.348	0.131	0.717	1.134 (0.573, 2.245)
C1 vs. C2					
Sharing the delivery room with other parturients during labor and delivery	−0.854	0.514	2.754	0.097	0.426 (0.155, 1.167)
Abnormal fetal heart rate during labor	−1.482	0.484	9.382	0.002	0.227 (0.088, 0.586)
Separation from the newborn during the puerperium	1.526	0.566	7.282	0.007	4.600 (1.518, 13.935)
Husband as the primary caregiver during the puerperium	2.097	0.801	6.847	0.009	8.141 (1.693, 39.157)
Breast engorgement severely affecting mood	−0.758	0.609	1.552	0.213	0.468 (0.142, 1.544)
Perineal wound pain lasting >2 days	−0.376	0.706	0.283	0.595	0.687 (0.172, 2.740)
Wound pain affecting daily life	1.173	0.492	5.682	0.017	3.233 (1.232, 8.483)

#### Association between latent classes and PTSD

The PTSD scores at 42 days postpartum showed a significant increase compared to those at 3 days postpartum for Class 1 and Class 2 (*P* < 0.05), whereas no statistically significant differences were observed between the two time points for Class 3 and Class 4 (*P* > 0.05). Furthermore, the magnitude of change in PTSD scores from 3 days to 42 days postpartum was significantly greater in Class 1 and Class 2 than in Class 3 and Class 4 (*P* < 0.05). The detailed results are presented in Table 7.

**Table 7:** Association between the four latent classes of childbirth trauma perception and postpartum PTSD.

PTSD	Class 1 (*n* = 41)	Class 2 (*n* = 62)	Class 3 (*n* = 14)	Class 4 (*n* = 52)	*F*	*P*
3 days postpartum	18 (17, 19)	19 (17, 26)	20 (18, 24)^a^	19 (18, 25.50)^a^	15.141	0.002
42 days postpartum	22 (20, 24)^d^	23 (20, 26)^d^	20 (18, 23)^a, b^	18 (17, 20.50)^a, b, c^	50.355	< 0.001
Difference	3 (2, 6)	3 (−2, 6)	0 (−3, 2)^a, b^	0 (−5, 0.50)^a, b^	52.342	< 0.001

Pairwise comparisons were performed with P-values adjusted using the Dunn-Bonferroni method. ^a^*P* < 0.05 compared with Class 1; ^b^*P* < 0.05 compared with Class 2; ^c^*P* < 0.05 compared with Class 3; ^d^*P* < 0.05 compared with scores at 3 days postpartum.

## Discussion

### Core findings and implications

Of note, because the latent classes were derived from trauma perception change scores over the same interval used to assess PTSD changes, these associations are exploratory and should not be interpreted as prospective prediction or causal inference.

This study applied latent profile analysis within a Chinese maternal population and identified four heterogeneous patterns of childbirth trauma perception: the Severe Childbirth Trauma Perception Group (C1), the High Childbirth Trauma Perception Group (C2), the Moderate Childbirth Trauma Perception Group (C3), and the Low Childbirth Trauma Perception Group (C4). Importantly, these four classes represent distinct patterns of childbirth trauma perception—a subjective appraisal of the childbirth experience—rather than successive stages of PTSD development. Although these perception patterns were differentially associated with subsequent PTSD symptoms, the cross-sectional nature of the class derivation and the short follow-up period preclude mechanistic or causal interpretations. This heterogeneity, as captured by the distinct patterns of scores across the four BTPS-WVD dimensions (Medical Support, Labor Pain, Family Support, and Childbirth Outcome), suggests that women may differ in the relative contribution of various aspects of the childbirth experience to their overall trauma perception. These distinct profiles provide a framework for understanding differential responses and implementing targeted interventions (19–21). More importantly, these distinct categories show systematic differentiation in their constituent influencing factors and developmental trajectories of PP-PTSD symptoms, offering crucial empirical evidence for shifting from a “one-size-fits-all” universal intervention to a “precision-based” risk stratification and prevention strategy. Unlike prior studies that focused on PTSD outcomes, we identified subgroups based on trauma perception, capturing subjective appraisals rather than clinical symptoms—which may explain differences in class distribution.

### Characteristics and connotations of latent classes of childbirth trauma perception

#### Clinical psychological profiles of each class

Consistent with previous research identifying intense pain and negative subjective experience as key correlates of traumatic birth (22–24), our C1 group scored highest on labor pain and childbirth outcome dimensions. The relatively lower scores on family support within C1 and C2 suggest that when medical and physical aspects are suboptimal, family support may serve as an important buffer (25). C3 (the moderate group), comprising 48.33% and being the largest group, scored slightly below average across dimensions, representing a “broad intermediate state” characterized by some negative feelings but overall manageable. In contrast, C2 (the high perception group, 20.67%) was classified as “high perception,” featuring above-average scores on multiple dimensions. This may indicate that its sources of trauma differ from C1, perhaps being more concentrated in dissatisfaction with the support system or specific medical procedures. Compared to C1 and C2, C3 and C4 had lower scores across dimensions and total scores, with flatter profile curves. However, their highest within-group score was on the “Family Support Trauma Perception” dimension. The existence of C4 (the low trauma group, 17.33%) is highly significant, demonstrating that achieving a low-trauma or even positive childbirth experience is possible. This group can be regarded as an ideal reference group possessing psychological resilience or protective resources. The construction of a positive birth experience primarily stems from the mother's self-confidence in her body's ability to cope with labor pain, mind-body coordination, and a dual support system from healthcare professionals and the spouse. Professional medical support and effective communication significantly enhance the mother's sense of control during labor, while a psycho-social support network strengthens her psychological resilience. These protective factors work synergistically to effectively buffer childbirth stress, reduce the risk of traumatic experiences, and, by promoting positive emotional experiences, establish long-term psychological protection mechanisms for the mother (26).

#### From static classes to dynamic change: the evolution of trauma perception

Results showed a significant increase in trauma perception levels at 42 days postpartum compared to 3 days postpartum, suggesting that maternal appraisal of the childbirth experience is not static but can evolve during the postpartum recovery period. This phenomenon may be closely related to trauma memory consolidation mechanisms and delayed stress responses (27–29). This finding emphasizes that the monitoring of childbirth trauma should extend beyond immediate postpartum assessment to focus on the potential developmental trajectory of trauma perception during the mid-to-late puerperium. However, the heterogeneity in individual change trends (some increasing, some decreasing) precisely corresponds to the different latent classes identified by LPA, suggesting that women in different classes may follow distinct psychological recovery paths. The observed heterogeneity in change trajectories suggests that the process of psychological adaptation to childbirth is not uniform. Individual differences in how women process and reappraise their birth experience over time, as reflected in the changing BTPS-WVD scores, may underpin these distinct developmental paths. For individuals with severe and high-perception childbirth trauma, professional interventions such as emotion-focused therapy and cognitive behavioral therapy can be employed to prevent progression to PTSD (30). Therefore, altering the developmental trajectory of childbirth trauma can be approached through perinatal management across the “antenatal-intrapartum-postpartum” continuum, social support, and postpartum continuity of care.

### Social support and its differential associations by source and timing

Multivariate logistic regression analysis revealed a complex network of factors influencing class membership, with some seemingly “paradoxical” findings warranting in-depth discussion. A multicenter cross-sectional study based on the ABC-X model in a Chinese maternal population showed that artificial labor induction, preterm birth, instrumental vaginal delivery, neurotic personality traits, and negative cognitions about the birth event were risk factors for childbirth trauma; perceived social support and care support were protective factors (31). This study provides a new pathway for the prevention and intervention of childbirth trauma from the perspective of “stressor-coping posture-perceived stress-crisis intervention,” suggesting targeted approaches focusing on reducing risk factors and enhancing protective factors.

#### Clinical events and their associations with trauma perception

This study found that “abnormal fetal heart rate during labor” was unexpectedly associated with a lower likelihood of belonging to the severe C1 group. While our data cannot confirm the underlying mechanisms, one speculative hypothesis for this unexpected association is that the heightened monitoring and clinical interventions triggered by a documented abnormality might paradoxically enhance maternal reassurance. This interpretation, however, requires direct testing in future studies designed to assess maternal perceptions of safety and provider communication during such events. Conversely, situations without clear medical indications but with more negative experiences (possibly stemming from unmet expectations or emotional neglect) may have more insidious and lasting psychological impacts. For mothers to achieve a positive birth experience, their core needs for feeling supported, in control, safe, and respected must be met (32). During childbirth, mothers are particularly sensitive to interactions with healthcare staff. Midwives and obstetricians have a critical impact on the mother's psychological state through effective communication and emotional support; positive interactions promote a positive birth experience, while negative interactions can lead to lasting psychological trauma and even increase the risk of PTSD (33). Therefore, professional management holds key value in buffering childbirth stress and reducing psychological trauma risk.

#### The dual faces of social support: the importance of source and context

The role of social support is not uniform. “Sharing the delivery room with other parturients during labor and delivery” was associated with a lower likelihood of belonging to the C1 group. One possible explanation is that the presence of peers may provide emotional resonance and normalize the childbirth experience. However, as peer support processes were not directly measured in this study, this interpretation remains speculative and requires further investigation. Peer support (including partners, family/friends, or trained companions) plays a bidirectional regulatory role in the prevention and recovery of childbirth trauma. Positive companionship can buffer intrapartum stress and reduce trauma risk by enhancing the mother's sense of control and security through emotional support, continuous encouragement, and information sharing. Conversely, if companions exhibit anxiety, helplessness, or inappropriate intervention, they may exacerbate the mother's sense of isolation and loss of control, becoming a potential catalyst for trauma formation. One study investigated the impact of companionless childbirth due to COVID-19 visitation bans on maternal mental health, finding that mothers who gave birth alone experienced higher psychological distress than those with companions, even affecting subsequent mother-infant bonding (34).

That “having the husband as the primary caregiver during the puerperium” was associated with a lower risk of belonging to the C2 group highlights the crucial value of practical and emotional care provided by core family members during the postpartum recovery period. From the results in Table 1, it can be inferred that the proportion of “husband as primary caregiver” in C2 is lower than in C3 and C1. The trend in Figure 2 shows that 2′s score on the “Family Support Trauma Perception” dimension is higher than that of C3 and C1. This fully demonstrates the effectiveness of partner care and support in reducing maternal childbirth trauma. Positive partner support can reduce trauma risk by enhancing the mother's sense of control during birth and perceived social support through emotional comfort, participation in decision-making, and physical care. Conversely, if the husband exhibits avoidance, anxiety, or insufficient support, it may exacerbate the mother's feelings of isolation and helplessness, becoming a potential factor in trauma formation (35). These findings collectively indicate that the “effectiveness” of social support is highly dependent on its source (peer or partner) and timing (intrapartum or puerperium). Insufficient partner involvement and gaps in emotional and informational support are significantly associated with the severity of postpartum childbirth trauma (36). Intervention measures should prioritize partner-involved care models and specifically address unmet informational and emotional needs.

#### Postpartum physical discomfort and its association with trauma perception

The analysis results consistently identified “breast engorgement severely affecting mood,” “perineal wound pain lasting >2 days,” and “wound pain affecting daily life” as stable and strong risk factors for belonging to the high-risk C1 and C2 classes. This strongly confirms that childbirth trauma does not end in the delivery room. Persistent physiological pain in the early postpartum period that affects daily functioning continually reminds and reinforces negative memories of childbirth, hindering psychological recovery and may hinder psychological recovery and reinforce negative childbirth memories. Interventional deliveries such as instrumental or emergency cesarean may trigger negative birth experiences and postpartum psychological distress. During childbirth, approximately 90% of women experience perineal trauma of varying degrees, with 3%−6% being severe and 13%−16% requiring episiotomy. Evidence indicates that perineal trauma can negatively impact the birth experience and postpartum mental health. Women often experience the perineal suturing process as painful and develop anxiety about future childbirth as a result (37, 38).

Breastfeeding has a dynamic association with postpartum childbirth trauma. Successful breastfeeding can exert a psychological protective effect by enhancing maternal role efficacy, strengthening mother-infant bonding, and alleviating anxiety through neuroendocrine mechanisms such as oxytocin release. Conversely, feeding difficulties (e.g., physiological engorgement, mastitis, or painful nursing) may activate existing childbirth trauma memories, exacerbating feelings of helplessness and self-doubt, leading to a continuation of the traumatic experience (6, 39).

Mother-infant separation during the puerperium is an important risk factor for childbirth trauma. This early separation disrupts the establishment of mother-infant bonding and may hinder the successful initiation of breastfeeding (40). Psychologically, separation deprives the mother of crucial early nurturing experiences, easily fostering feelings of loss of control, helplessness, and negative evaluations of the birth process, thereby reinforcing traumatic memories. Physiologically, it interferes with normal postpartum neuroendocrine regulation and adaptation (41). Therefore, mother-infant separation may not only directly cause psychological distress but also significantly increase the mother's risk of developing postpartum traumatic stress symptoms, making it a core issue requiring focused attention in the prevention and intervention of childbirth trauma. This study confirms that “separation from the newborn during the puerperium” is a risk factor for severe childbirth trauma perception (C1), consistent with previous research conclusions.

Therefore, postpartum continuity of care services—through systematic pain management, lactation support, and wound care—not only promotes physical recovery but is also a key psychological intervention for preventing childbirth trauma. These measures can effectively interrupt the mutual reinforcement between postpartum complications and psychological trauma, helping mothers establish positive parenting experiences and a stable maternal role identity. Emphasizing a sense of control and quality of care during childbirth is key to promoting positive birth experiences. The quality of intrapartum care and the supportive relationships established during labor are more important than mere presence or service continuity. On the foundation of ensuring high-quality intrapartum interaction, promoting the implementation of postpartum continuity of care is significant for consolidating the long-term effects of positive birth experiences and promoting maternal psychological and physical adaptation (22).

### Association between latent classes and changes in postpartum PTSD symptoms

It should be noted, however, that the present findings reflect associations between trauma perception classes and PTSD symptom changes, and do not establish that one construct necessarily develops into the other. Rather, childbirth trauma perception represents a subjective appraisal that may inform subsequent symptom risk, but the pathways linking these constructs require further investigation.

Unlike previous person-centered studies that identified profiles based on PTSD symptom severity at single or multiple time points, our LPA was based on change scores in trauma perception, thereby capturing patterns of change rather than static severity levels—a distinction with potential clinical implications for targeted intervention. Longitudinal data indicate that LPA classification based on early postpartum trauma perception patterns is associated with subsequent psychological outcomes. High-trauma-perception groups (C1 and C2) exhibited a significant increase in PTSD symptoms at 42 days postpartum compared to 3 days postpartum (*P* < 0.05), suggesting that for these mothers, psychological stress responses may not naturally dissipate over time. This aligns with the vicious cycle mechanism in trauma theory involving “persistent hyperarousal” and “recurrent intrusive traumatic memories.” Research shows that approximately 30% of postpartum women are troubled by psychological childbirth trauma, some of whom may further develop elevated traumatic stress symptoms, even reaching the screening threshold for PTSD (42). These findings suggest a potential link between early trauma perception patterns and subsequent PTSD symptom changes that merits further investigation in prospective studies with independent samples. Low-trauma-perception groups (C3 and C4) showed no significant change in PTSD scores between the two time points (*P* > 0.05), and the magnitude of symptom change was significantly lower than in the high-perception groups, indicating a psychological recovery trajectory more aligned with natural adaptation. The worsening symptoms over time in the high-perception group suggest a risk of persistent symptom elevation, while the stability in the low-perception group reflects a natural recovery trend. This suggests that trauma perception patterns may be associated with the course of PTSD symptoms, although prospective studies are needed to confirm this relationship. This is consistent with Horsch et al.'s (11) conclusion that childbirth-related PTSD symptoms are closely linked to traumatic birth experiences. Latest data indicate that up to 45% of mothers report experiencing childbirth trauma, and PTSD symptoms resulting from childbirth trauma occur in approximately 1.5 to 9% of all births (43). Risk factors for childbirth-related PTSD symptoms exhibit stage-specific characteristics (11). Prenatal factors primarily include depression, fear of childbirth, health problems, and trauma history. Core intrapartum risks encompass negative subjective experience, operative delivery, and dissociative states, while medical support is an important protective factor. Postpartum factors are associated with comorbid depression, persistent stress, and poor coping. Good medical support during labor is key to improving birth outcomes and reducing PTSD symptom risk.

### Research contributions, limitations, and future directions

#### Theoretical contributions and practical implications

Theoretically, this study adopts latent profile analysis and an “individual-centered” research perspective, providing a more refined framework for studying psychological responses to childbirth trauma in the Chinese cultural context, emphasizing its multidimensional and heterogeneous nature.

Practically, the study results provide a “three-stage” intervention pathway covering the perinatal period for clinical care. During pregnancy, protective factors can be frontloaded. Healthcare institutions can offer pregnant women rich antenatal classes, group communication activities, ward visits, and one-on-one consultations to help them build childbirth knowledge, confidence, and trust in the medical institution. During childbirth, the delivery room environment should be optimized to promote peer support (e.g., establishing family-centered birthing rooms, encouraging partner presence), and effective intrapartum communication between healthcare providers and mothers should be strengthened, respecting maternal needs, safeguarding their right to informed consent, and enhancing their sense of control. During the puerperium, comprehensive postpartum rehabilitation and continuity of care should be provided, with a core focus on alleviating postpartum physical discomfort. While ensuring medical safety, physical separation of mother and infant should be minimized to promote early contact and breastfeeding. For situations where separation is necessary, timely clear communication, emotional support, and visitation conditions should be provided, such as establishing Mother-Newborn Care Units (MNCUs) (44). Concurrently, trauma-informed care should be implemented to identify high-risk mothers, and family support networks should be actively mobilized. Postpartum assessment should be timely, focusing on the heterogeneity of trauma perception to early identify mothers with C1/C2 characteristics. Comprehensive, intensive psychological interventions (e.g., trauma-focused cognitive behavioral therapy) should be implemented for the C1 group, and targeted preventive support (e.g., pain management, breastfeeding counseling, building family-social support chains) should be provided for the C2/C3 groups.

### Limitations and future directions

This study has several limitations. First, the single-center sample limits the generalizability of the results, and our exclusion criteria further restricted the sample to relatively low-risk women. Second, some data were retrospective self-reports, which may be subject to recall bias. Our exclusion of several established PTSD risk factors limits generalizability, and the observed class distribution may not represent higher-risk populations. Third, the study did not include all potential influencing factors, such as maternal personality traits, childhood trauma history, and specific birth expectations. Fourth, the predictor selection procedure was data-driven (univariate screening followed by stepwise regression), which may be susceptible to overfitting and model-selection bias. Although we reported 95% CIs and emphasized the magnitude of associations, the results are exploratory and require external validation. Fourth, the PCL-C is based on DSM-IV criteria. While it remains a widely used and reliable measure of PTSD symptom severity in perinatal research, it is not a diagnostic tool. We acknowledge that the DSM-5-based City BiTS, now validated in Chinese populations, represents a more contemporary measure for future research. Additionally, structured clinical interviews such as the CAPS-5 would strengthen diagnostic validity in subsequent studies. Fifth, while the present study identified patterns of change in trauma perception, future research should examine whether these change patterns are stable over longer follow-up periods and how they relate to PTSD symptom profiles identified in previous person-centered studies. Additionally, because the latent classes were derived from change scores over the same interval used to assess PTSD changes, these associations are exploratory and not prospective or causal. The four-class solution requires replication in independent samples before clinical application. Finally, several speculative mechanisms proposed for observed associations were not directly tested and require future investigation.

Future research could improve in the following aspects: (1) Conduct multicenter, large-sample longitudinal studies to test the stability of the latent classes and their impact on longer-term maternal and infant outcomes. (2) Investigate the specific mechanisms of protective factors (e.g., “sharing the delivery room”) and the physiological-psychological pathways through which risk factors (e.g., postpartum pain) affect psychological state. (3) Based on the class characteristics and risk factors identified in this study, develop and validate stepped psychological intervention programs tailored to mothers at different risk levels.

## Conclusion

In summary, this study identified four heterogeneous classes of childbirth trauma perception among Chinese parturients, each possessing distinct clinical features, compositions of influencing factors, and associations with PTSD developmental trajectories. Persistent postpartum physical discomfort is a core risk factor exacerbating trauma perception, whereas timely professional management and specific forms of social support may buffer severe trauma perception. This suggests that the focus of childbirth psychological care should be shifted forward to the prenatal period and extended backward into the postpartum recovery phase. By early identification of high-risk classes and implementing targeted interventions, it is possible to effectively prevent the worsening of childbirth trauma perception and its progression to PTSD, thereby enhancing maternal and infant mental health.

## Data Availability

The raw data supporting the conclusions of this article will be made available by the authors, without undue reservation.
